# Research on community-based health workers is needed to achieve the sustainable development goals

**DOI:** 10.2471/BLT.16.185918

**Published:** 2016-11-01

**Authors:** Dermot Maher, Giorgio Cometto

**Affiliations:** a Special Programme for Research and Training in Tropical Diseases, World Health Organization, avenue Appia 20, 1211 Geneva 27, Switzerland.; b Health Workforce Department, World Health Organization, Geneva, Switzerland.

“Leaving no-one behind” is a rallying call to remind us of the need to ensure everybody benefits from the drive to achieve the *2030 Agenda for sustainable development goals* (SDGs). Building on the considerable progress made regarding the millennium development goals, there is a need for a comprehensive and coordinated effort to scale up effective interventions.^1^ Achieving universal health coverage and scale up will require systems thinking that maximizes the potential of using all parts of the health system to ensure equitable delivery of curative and preventive services. The *WHO Global strategy on human resources for health: workforce 2030* recognizes that addressing population needs for the SDGs requires a more sustainable and responsive skills mix, harnessing the potential of community-based health workers in inter-professional primary care teams, and calling for the integration of these cadres in the health system.^2^

The contribution of community-based health workers is relevant to most service delivery priorities at the primary health care level, particularly in underserved areas. Community-based health workers are key partners in health care delivery and play a critical role in promoting equitable expansion of coverage for a range of preventive, promotive and curative services related to reproductive, maternal, newborn and child health, ^3^^–^^6^ infectious diseases^7^ and noncommunicable diseases.^8^^,^^9^ Systematic appraisal of the current evidence on the effectiveness of community-based health workers in delivering these services is important in developing guidance on health policy and system support that focuses on optimizing the use of community-based health workers for universal health coverage.^10^ The identification of gaps in existing knowledge should inform a future research agenda.

The investment case for community-based health workers requires strong evidence that is based on good quality research. We identify five key issues for consideration in building this evidence base. 

First, while there is a wealth of research experience on the role of community-based health workers regarding communicable diseases and maternal and child health, there is less research on their role regarding noncommunicable diseases, which are responsible for an increasing proportion of the global burden of disease. Given that many countries are going through an epidemiologic transition, it will be critical to define research and policy priorities, with flexibility to allow countries to adapt them to their population and health system needs over time. 

Second, more attention should be paid to cross-cutting enabling factors, for example, education, accreditation and regulation, management and supervision, effective linkage to professional cadres, motivation and remuneration, and provision of essential drugs and commodities. 

Third, there is a research gap in understanding how to ensure the sustainability of programmes supported by community-based health workers, by using innovative national planning, governance, legal and financing mechanisms.

Fourth, previous research experience on the role of community-based health workers represents a mix of varying degrees of quality, while the emphasis of future research must be on scientific rigour to strengthen the evidence base for policy and practice. 

Finally, it is important to avoid too narrow a disease- or intervention-specific focus to community-based health workers’ research. There is a need to investigate not only the effectiveness question (what works), but also the contextual factors and enablers (how, for whom, under what circumstances).^11^ Getting an answer to such policy questions will require research that uses mixed methods.

As we strive to fulfil the promise of the SDGs and ensure we “leave no-one behind”, it is important that we seize the opportunity to promote research on community-based health workers and their potential contribution to public policy objectives. The considerable experience of WHO and special programmes such as the Special Programme for Research and Training in Tropical Diseases in promoting quality research on community-based health workers provides a strong platform to identify the knowledge gaps and research needs in building the evidence base.
